# Genome-wide identification and expression analysis of *PtJAZ* gene family in poplar (*Populus trichocarpa*)

**DOI:** 10.1186/s12863-023-01150-5

**Published:** 2023-09-21

**Authors:** Gaixia Yang, Shijie Wang, Lianxiang Long, Xiaoyue Yu, Hongyu Cai, Pengyun Chen, Lijiao Gu, Minsheng Yang

**Affiliations:** 1 Forest Department, Forestry College, Hebei Agricultural University, Baoding, China; 2 Hebei Key Laboratory for Tree Genetic Resources and Forest Protection, Baoding, 071000 China; 3 College of Landscape Architecture and Tourism, Hebei Agricultural University, Baoding, China; 4 State Key Laboratory of Cotton Biology, School of Life Sciences, College of Agriculture, Henan University, Kaifeng, 475004 China

**Keywords:** *Populus trichocarpa*, *JAZ* gene family, Bioinformatics analysis, Expression patterns, Functional prediction

## Abstract

**Background:**

The jasmonate ZIM domain (JAZ) protein is a key repressor of the jasmonate signal transduction pathway, which plays an important role in plant growth and development and defense responses. In this study, based on the published whole-genome data, we identified members of the *JAZ* gene family in *Populus trichocarpa*. Through a series of bioinformatic approaches, their expression patterns under various stress conditions have been analyzed to explore and excavate the endogenous resistance genes of poplar and provide a theoretical basis for breeding new varieties of poplar resistance.

**Results:**

A total of 13 *PtJAZ* genes have been identified in *P. trichocarpa* and designated as *PtJAZ1*–*PtJAZ13*. Those 13 *PtJAZ* genes were unevenly distributed on nine chromosomes, and they could be divided into four subfamilies. The gene structures and motif composition of the members derived from the same subfamily were similar. Collinearity analysis demonstrated that, compared with *Arabidopsis thaliana* and *Oryza sativa*, the most collinear pairs (13) were found in *P. trichocarpa* and *Eucalyptus robusta*. *Cis*-acting element analysis suggested that the promoter regions of *PtJAZs* contained a large number of hormones and stress response elements, of which abscisic acid (ABA) and methyl jasmonate (MeJA) hormone response elements were the most abundant. The *PtJAZ* genes not only had diverse expression patterns in different tissues, but they also responded to various abiotic and biotic stress conditions. The co-expression network and GO and KEGG analyses showed that *JAZ* genes were closely related to insect resistance.

**Conclusions:**

In this study, applying bioinformatic methods, 13 *PtJAZ* gene family members from *P. trichocarpa* were identified and comprehensively analyzed. By further studying the function of the poplar *JAZ* gene family, the aim is to select genes with better insect resistance and stress resistance so as to lay a solid foundation for the subsequent breeding of new poplar varieties.

**Supplementary Information:**

The online version contains supplementary material available at 10.1186/s12863-023-01150-5.

## Background

Jasmonate acid (JA) is an endogenous growth regulator in higher plants and participates in regulating most of the physiological processes, including plant growth and development [1]. There are two main JA derivatives: jasmonate-amino acid (JA-Ile) and MeJA [1]. JAZ protein family members play a broad role in plant development and defense responses. They also represent key repressors in the JA signal transduction pathway [2]. JAZs belong to the TIFY family, which includes four subfamilies: JAZ, ZML, PPD, and TIFY [3]. JAZs are repressor proteins composed of two main conserved domains, with TIFY (also known as ZIM) and Jas (also known as CCT-2) functional domains at the N- and C-terminus, respectively [4]. The JA pathway mainly includes three components: COI1, the SCF^COI1^ complex, and the JAZ protein [5]. COI1 is a 66 kDa F-box protein that is a JA receptor and plays a critical role in the jasmonic signal transduction pathway [5]. The SCF^COI1^ complex can bind the E3 ubiquitin ligase and be ubiquitinated by JAZ to regulate the expression of JA-responsive genes. Furthermore, similar to the function of COI1, the JAZ protein is also a receptor in the jasmonate signaling pathway [6].

In recent years, with the rapid development of genome sequencing technology, an increasing number of plant *JAZ* genes have been characterized. For example, 12 *JAZ* genes were identified in the *P. trichocarpa TIFY* gene family [3, 7]. Nine, 12, 43, 26, 16, and 17 *JAZ* genes were found in *Aegilops taushii* [8], *Camellia Sinensis* [9], *Ipomoea batatas* [10], *Solanum lycopersicum* [11], *Zea mays* [12], and *Juglans regia L.* [13], respectively. Moreover, using Y2H technology, Li et al. [14] constructed a *Solanum lycopersicum* JAZ interaction network that contained 13 members. Previous studies have shown that *JAZ* genes play an important role in the growth, development, and stress defense responses of plants. Zhang et al. identified 11 *JAZ* genes from the *Vitis vinifera* genome and found that their expression levels changed significantly under different abiotic stress conditions (e.g., drought, cold, and salt) and hormone treatments (e.g., JA and ABA) [15]. Thirty *GhJAZ* genes were identified in *Gossypium hirsutum*, and the results demonstrated that they may regulate fiber differentiation and development by interacting with cotton fiber initiation factors [16]. Wu et al. characterized the phenotypes of overexpressed *OsJAZ1* and *OsJAZ9* transgenic rice lines at the seedling stage and found obvious phenotypes relating to drought and salt stress, respectively [17]. Yu et al. studied the expression patterns of 15 *JAZ* genes in *Prunus persica*. Among which, three members (e.g., *PpJAZ1*, *4*, and *5*) may be stimulated by MeJA and positively correlated with the process of exocarp pigmentation [18]. Except for responding to abiotic stresses, the JAZ protein may also function in anti-insect. For example, the expression levels of three *Toona ciliata JAZ* genes (e.g., *TciJAZ1*, *3*, and *11*) were significantly upregulated in the leaves and tender stems under *Hypsipyla robusta Moore* (a borer pest of Meliaceae) stress [19].

Poplar is the main worldwide afforestation tree species, especially in northern China. Poplar is an important model plant for the study of genetic engineering of forest trees, and it is also the first perennial woody plant for which the whole genome sequence has been determined [20]. In consideration of the fact that the *JAZ* gene family plays an important role in the growth and development of the plant kingdom. In this study, based on the published whole genome of *P. trichocarpa*, using bioinformatics approaches, a total of 13 *PtJAZ* members were identified at the chromosomal level. One more gene than Wang and Xia et al. [3, 7], in addition to We also profiled their transcriptional landscape between different tissues, abiotic and biotic stress conditions, and hormone treatments. There are also co-expression networks and GO and KEGG analyses, which Wang and Xia et al. [3, 7] do not have. Our data will provide a reference for subsequent research on poplar resistance genes.

## Results

### Identification and analysis of the *PtJAZ* gene family in *Populus trichocarpa*

**Fig. 1:**
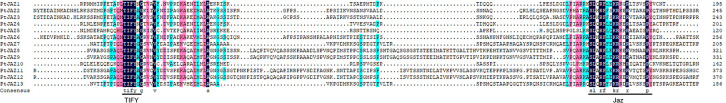
Multiple alignment of JAZ proteins from *Populus trichocarpa* Note: The TIFY and Jaz domain were indicated by black boxes

The *PtJAZ* gene family members were identified from the *P. trichocarpa* genome (v4.1) using hidden Markov models of the Jaz and TIFY domains. After verification of protein domains, a total of 13 *PtJAZ* genes were screened and named *PtJAZ1*-*PtJAZ13*. The amino acid sequence alignment analysis of these 13 PtJAZ proteins revealed that all members contained two canonical Jaz and TIFY domains (Fig. 1). The basic information analysis of the family members shows that the number of inner and outer membrane transmembrane helices of the 13 PtJAZ proteins is 0, and there is no transmembrane region, so it is predicted that they are not membrane proteins. The number of signal peptides is 0, indicating that there is no signal peptide in this family of proteins and that it is a nonsecreted protein. The predicted subcellular localization results showed that all 13 *PtJAZ* genes were located in the nucleus, and the number of exons of these family members is from 2 to 7, while the number of introns is from 1 to 6. In addition, the secondary structure of PtJAZ family proteins is mainly composed of random coils (51.68-73.96%), α-helices (11.22-32.21%), extended strands (7.43-14.36%), and β-turns (2.60-5.64%). PtJAZ11 and PtJAZ12 differed from the rest in that random coil > extended chain > α-helix > β-turn, whereas for the others, random coil > α-helix > extended chain > β-turn (Table 1).

### Chromosomal location of *PtJAZ* family genes in poplar

**Table 1:** The information of JAZ genes from *Populus trichocarpa*

Gene Name ^a^	Gene Name ^b^	Gene ID	Chromosome	Exon	Intron	Subcellular localization	Signal peptide	α-helix (%)	Extended chain (%)	β-turn (%)	Random coil (%)	Number of transmembrane
*PtJAZ1*	*PtJAZ1*	Potri.001G062500	Chr01	6	5	Nuclear	0	25.64	9.74	5.64	58.97	0
*PtJAZ2*	*PtJAZ2*	Potri.001G166200	Chr01	5	4	Nuclear	0	20.45	7.43	2.60	69.52	0
*PtJAZ3*	*PtJAZ3*	Potri.003G068900	Chr03	5	4	Nuclear	0	20.60	8.61	4.12	66.67	0
*PtJAZ4*	*PtJAZ4*	Potri.003G165000	Chr03	6	5	Nuclear	0	31.34	9.95	4.98	53.73	0
*PtJAZ5*	-	Potri.006G023301	Chr06	3	2	Nuclear	0	31.67	11.67	3.33	53.33	0
*PtJAZ6*	*PtJAZ5*	Potri.006G139400	Chr06	5	4	Nuclear	0	15.69	8.39	2.92	72.99	0
*PtJAZ7*	*PtJAZ6*	Potri.006G217200	Chr06	5	4	Nuclear	0	27.54	8.47	3.39	60.59	0
*PtJAZ8*	*PtJAZ7*	Potri.008G133400	Chr08	7	6	Nuclear	0	13.72	9.76	3.05	73.48	0
*PtJAZ9*	*PtJAZ8*	Potri.010G108200	Chr10	7	6	Nuclear	0	13.31	9.76	2.96	73.96	0
*PtJAZ10*	*PtJAZ9*	Potri.011G083900	Chr11	2	1	Nuclear	0	32.21	10.74	5.37	51.68	0
*PtJAZ11*	*PtJAZ10*	Potri.012G044900	Chr12	7	6	Nuclear	0	11.22	14.29	3.06	71.43	0
*PtJAZ12*	*PtJAZ11*	Potri.015G035800	Chr15	7	6	Nuclear	0	12.34	14.36	2.77	70.53	0
*PtJAZ13*	*PtJAZ12*	Potri.018G047100	Chr18	5	4	Nuclear	0	23.96	10.60	3.23	62.21	0

Note: Gene name^a^ is the name of *PtJAZ* gene family members identified in this study. Gene name^b^ is the name of *PtJAZ* gene family members identified by Wang et al. and Xia et al. [3, 7]

### Chromosomal location of *PtJAZ* family genes in *Populus trichocarpa*

**Fig. 2:**
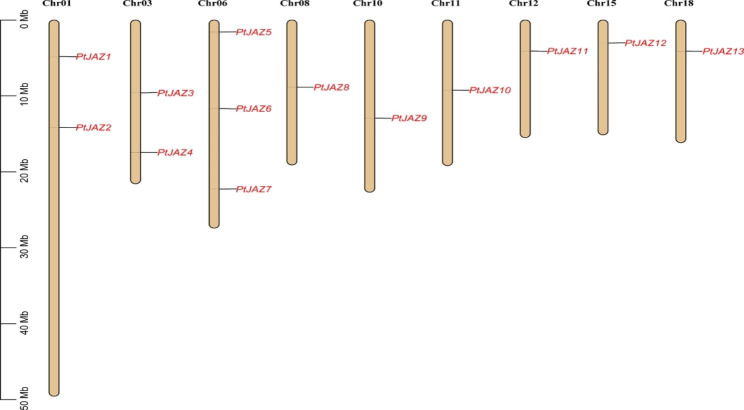
Chromosomal distribution of *PtJAZ* family genes

The chromosomal location distribution map was drawn according to the locations of the *PtJAZ* genes on the poplar genome. Figure 2 shows that 13 *PtJAZ* genes are distributed on 9 chromosomes, and the remaining 10 chromosomes, Chr02, Chr04, Chr05, Chr07, Chr09, Chr13, Chr14, Chr16, Chr17, and Chr19, have no *PtJAZ* gene distribution. Chr01 has 2 *PtJAZ* genes (*PtJAZ1* and *PtJAZ2*), Chr03 has 2 *PtJAZ* genes (*PtJAZ3* and *PtJAZ4*), Chr06 has 3 *PtJAZ* genes (*PtJAZ5*, *PtJAZ6*, and *PtJAZ7*), and the remaining chromosomes each have only one *PtJAZ* gene.

### Phylogenetic tree analysis of the *JAZ* family in various species

**Fig. 3:**
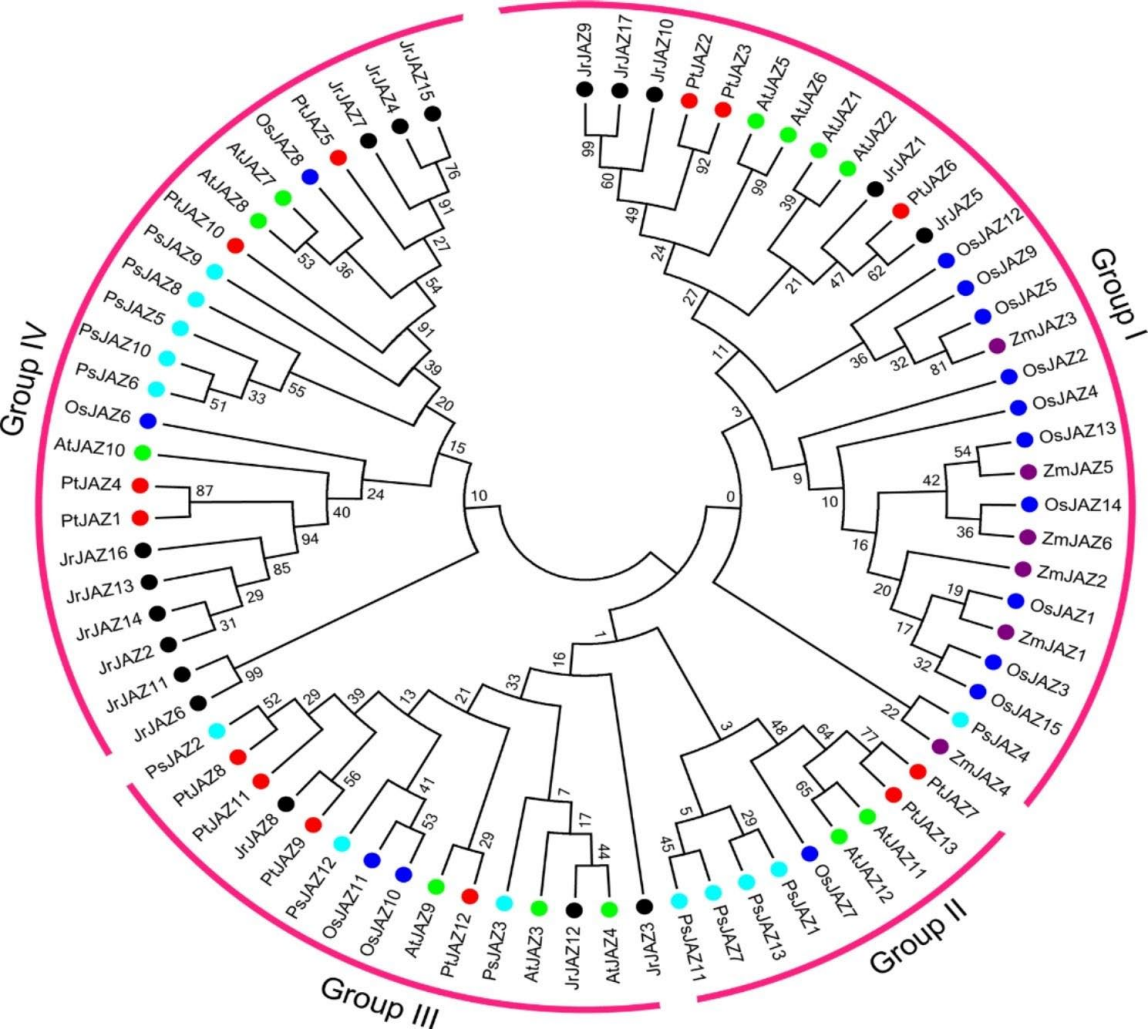
Phylogenetic tree of JAZ family proteins from *Populus trichocarpa* (*Pt*), *Arabidopsis thaliana* (*At*), *Oryza sativa* (*Os*), *Zea mays* (*Zm*), *Juglans regia* (*Jr*) and *Picea sitchensis* (*Ps*). Different colored dots in the phylogenetic tree represent JAZ proteins from different species

To further understand the evolutionary relationship of the *PtJAZ* family, the *P*.*trichocarpa* (13), *Arabidopsis thaliana* (12), *Oryza sativa* (15), *Zea mays* (6), *Juglans regia* (17), and *Picea sitchensis* (13) JAZ protein sequences were used to construct a phylogenetic tree. The results showed that the *PtJAZ* gene family can be divided into four subfamilies, among which subfamily II contained the least number of *PtJAZ* genes, while subfamilies III and IV contained the most *PtJAZ* genes. Subfamily II includes two genes, *PtJAZ7* and *PtJAZ13*; subfamily I includes three genes, *PtJAZ2*, *PtJAZ3*, and *PtJAZ6*; subfamily III includes four genes, *PtJAZ8*, *PtJAZ9*, *PtJAZ11*, and *PtJAZ12*; and subfamily IV includes four genes, *PtJAZ1*, *PtJAZ4*, *PtJAZ5*, and *PtJAZ10* (Fig. 3). Interestingly, all *ZmJAZ* genes are only distributed in subfamily I, and 10 genes in *OsJAZ* (15) are also distributed in this group. The *PsJAZ* gene was distributed in all four subfamilies, and most of the *PtJAZ* genes had higher homology with *Arabidopsis thaliana* and *Juglans regia.*.

### Gene structure and conserved motif analysis of the *PtJAZ* family in *Populus trichocarpa*

**Fig. 4:**
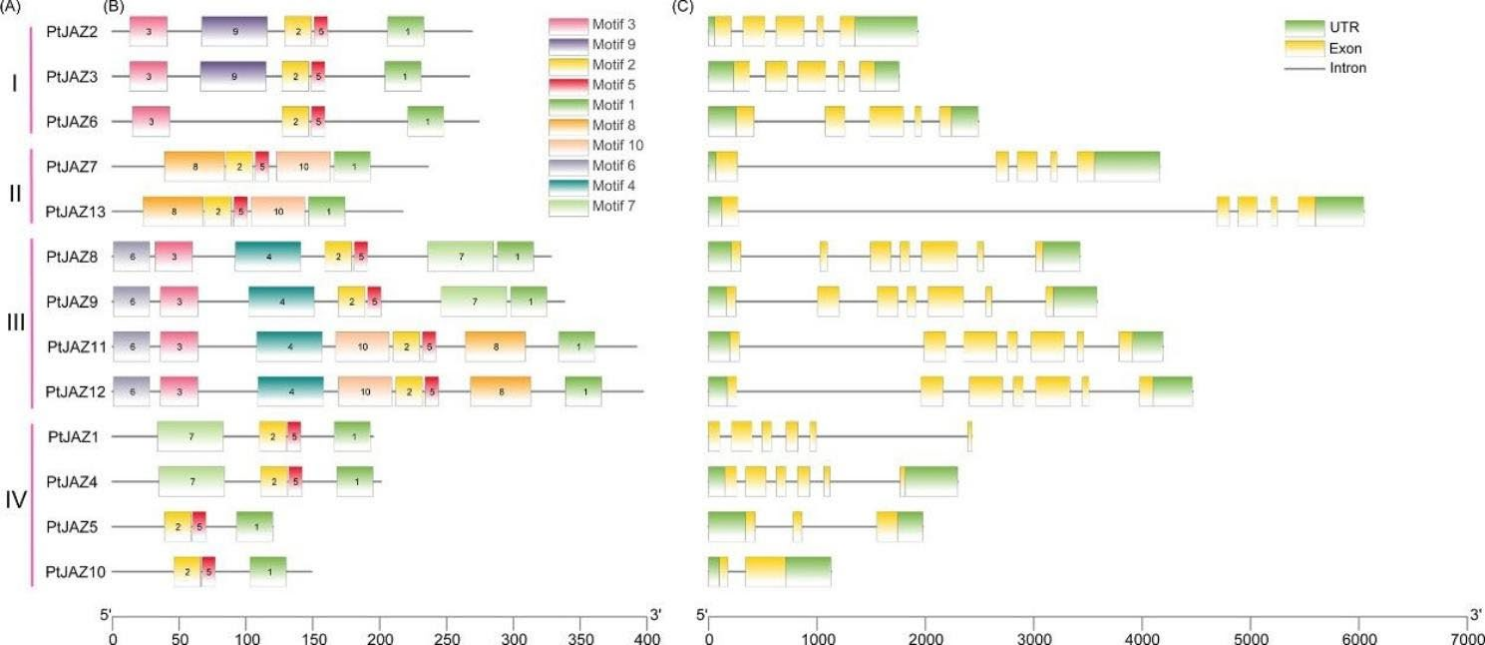
Gene structure and conserved motif analysis of *PtJAZ* family genes

The gene structure and motif distribution of *PtJAZ* family genes were drawn using the online software GSDS and MEME. Figure 4 A shows members of different subfamilies of *PtJAZ* genes. Conserved motif analysis revealed that most of the PtJAZ proteins contain similar motif types and orderings (Fig. 4B). Three conserved motifs, motif 1, motif 2, and motif 5, are present in all PtJAZs. The first subfamily members contain five motifs: motif 1, motif 2, motif 3, motif 5, and motif 9. The members of subfamily II all contain motif 1, motif 2, motif 5, motif 8, and motif 10. The members of subfamily III all contain motifs 1–6, with PtJAZ8 and PtJAZ9 also containing motif 7, with PtJAZ11 and PtJAZ12 also containing motif 8 and motif 10. Subfamily IV members all contain motif 1, motif 2, and motif 5, with both PtJAZ1 and PtJAZ4 containing motif 7 (Fig. 4B). The gene structure is shown in Fig. 4C, which shows that the members of subfamily I, *PtJAZ2*, *PtJAZ3*, and *PtJAZ6*, contain 5 exons and 4 introns. Subfamily II members *PtJAZ7* and *PtJAZ13* contain 5 exons and 4 introns. Subfamily III members all contain 7 exons and 6 introns. Subfamily IV members *PtJAZ1* and *PtJAZ4* contain 6 exons and 5 introns, *PtJAZ5* genes contain 3 exons and 2 introns, and *PtJAZ10* genes contain 2 exons and 1 intron. The analysis of the results shows that different *JAZ* gene subfamily members have similar motifs and gene structures, indicating that they have a relatively recent evolutionary relationship. However, there are some differences within subfamilies, indicating that the genes of the same subfamily members may also have functional diversity.

(**A**) Subfamily classification of *PtJAZ* family members. (**B**) Ten motifs of PtJAZ proteins analyzed by MEME software. (**C**) Gene structure of the *PtJAZ* genes. The UTR, introns, and exons are indicated with green boxes, black lines, and yellow boxes, respectively.

### Collinear analysis of *JAZ* genes in different species

**Fig. 5:**
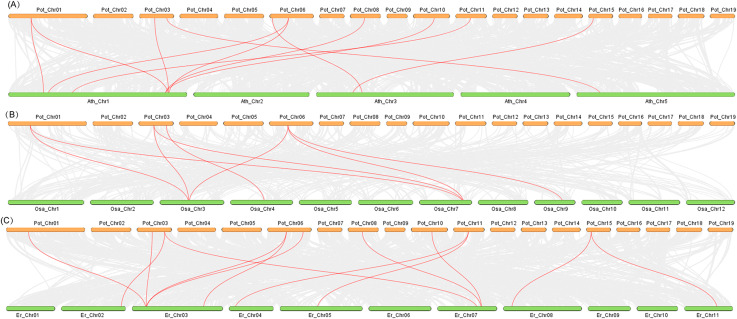
Collinear analysis of *PtJAZ* genes from *Populus trichocarpa* with *Arabidopsis thaliana*, *Oryza sativa* and *Eucalyptus robusta*. (**A**) Collinear analysis of *PtJAZ* genes with *Arabidopsis thaliana*. (**B**) Collinear analysis of *PtJAZ* genes with *Oryza sativa*. (**C**) Collinear analysis of *PtJAZ* genes with *Eucalyptus robusta*

The *PtJAZ* genes in poplar and the *Arabidopsis thaliana*, *Oryza sativa*, and *Eucalyptus robusta* genomes were analyzed for collinearity using TBtools. As shown in Fig. 5 and 11 collinear gene pairs were found in *P. trichocarpa* and *Arabidopsis*; 8 collinear gene pairs were found in *P. trichocarpa* and *Oryza sativa*; and 13 collinear gene pairs were found in *P. trichocarpa* and *Eucalyptus*, indicating that the *JAZ* genes in *P. trichocarpa* and *Eucalyptus robusta* have a more recent evolutionary relationship.

### Promoter *cis*-element analysis of the *PtJAZ* genes family

**Fig. 6:**
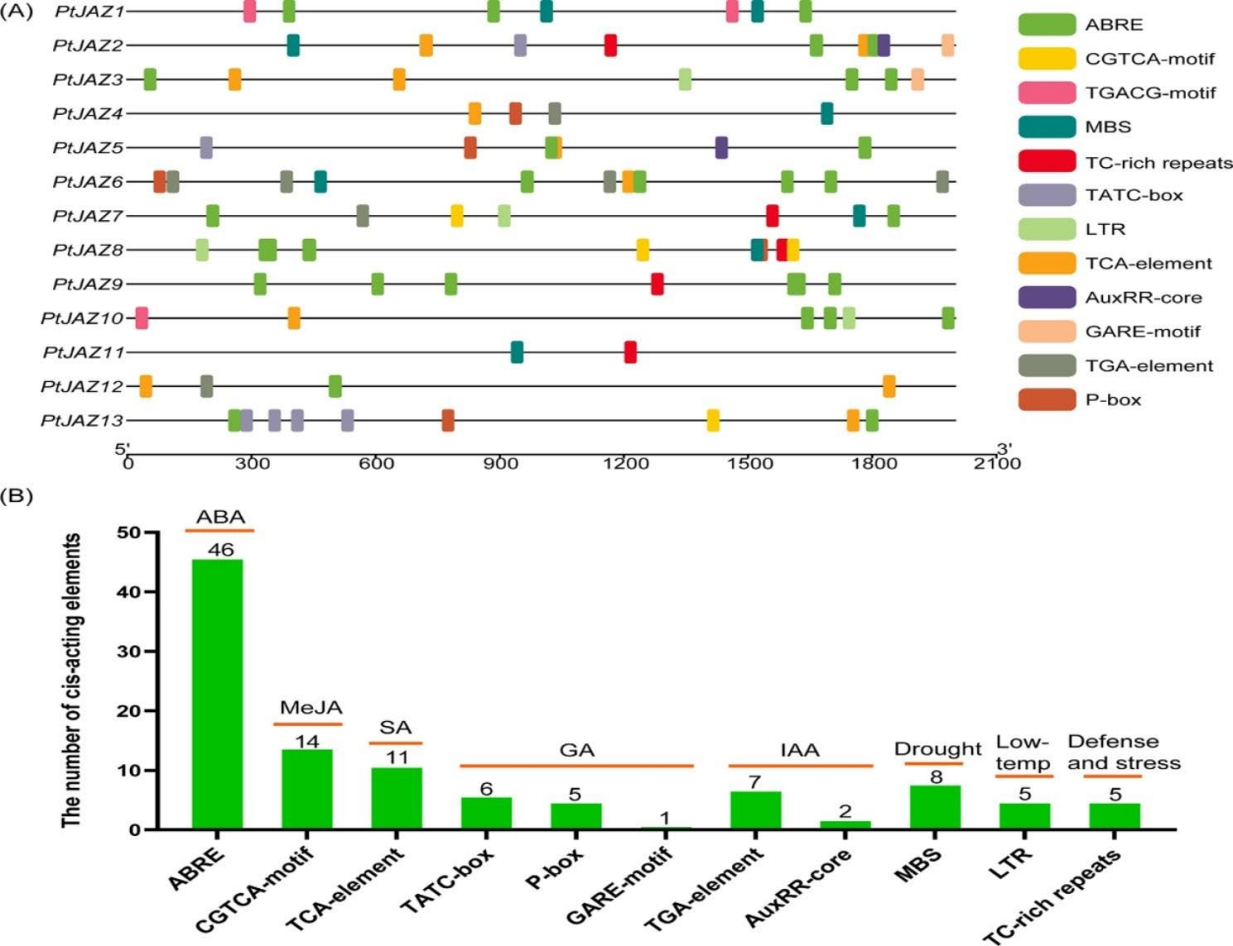
Analysis of *cis*-elements in the 2000 bp upstream promoter of *PtJAZ* family genes. (**A**) Distribution of *cis*-acting elements in the *PtJAZ* promoters. (**B**) Statistics of *cis*-acting elements in the *PtJAZ* promoters

The upstream 2000-bp promoter sequences of *PtJAZ* family genes were extracted from the poplar genome and submitted to PlantCARE online software to predict *cis*-acting elements. The distribution of *cis*-acting elements in the *PtJAZ* gene promoters is shown in Fig. 6A. As can be seen from the figure, the promoter region of each gene contains stress-related *cis*-acting elements, of which the number of *PtJAZ6* is up to 11, mainly auxin (TGA-motif) response elements and ABA response elements (ABRE), and the number of *PtJAZ11* is at least 2. As can be seen from Fig. 6B, these *cis*-acting elements include hormone response elements such as ABA, MeJA (CGTCA-motif, TGACG-motif), gibberellin (GA) (GARE-motif, P-box, TATC-motif), Auxin (IAA), salicylic acid (SA) (TCA-element), and stress response elements such as low temperature, drought, and resistance. Among them, the number of ABA response elements (46) and MeJA response elements (14) was the highest, suggesting that *PtJAZ* genes may be involved in plant resistance through ABA and MeJA signaling pathways.

### Expression patterns of *PtJAZ* family genes in various tissues and under various abiotic stresses

**Fig. 7:**
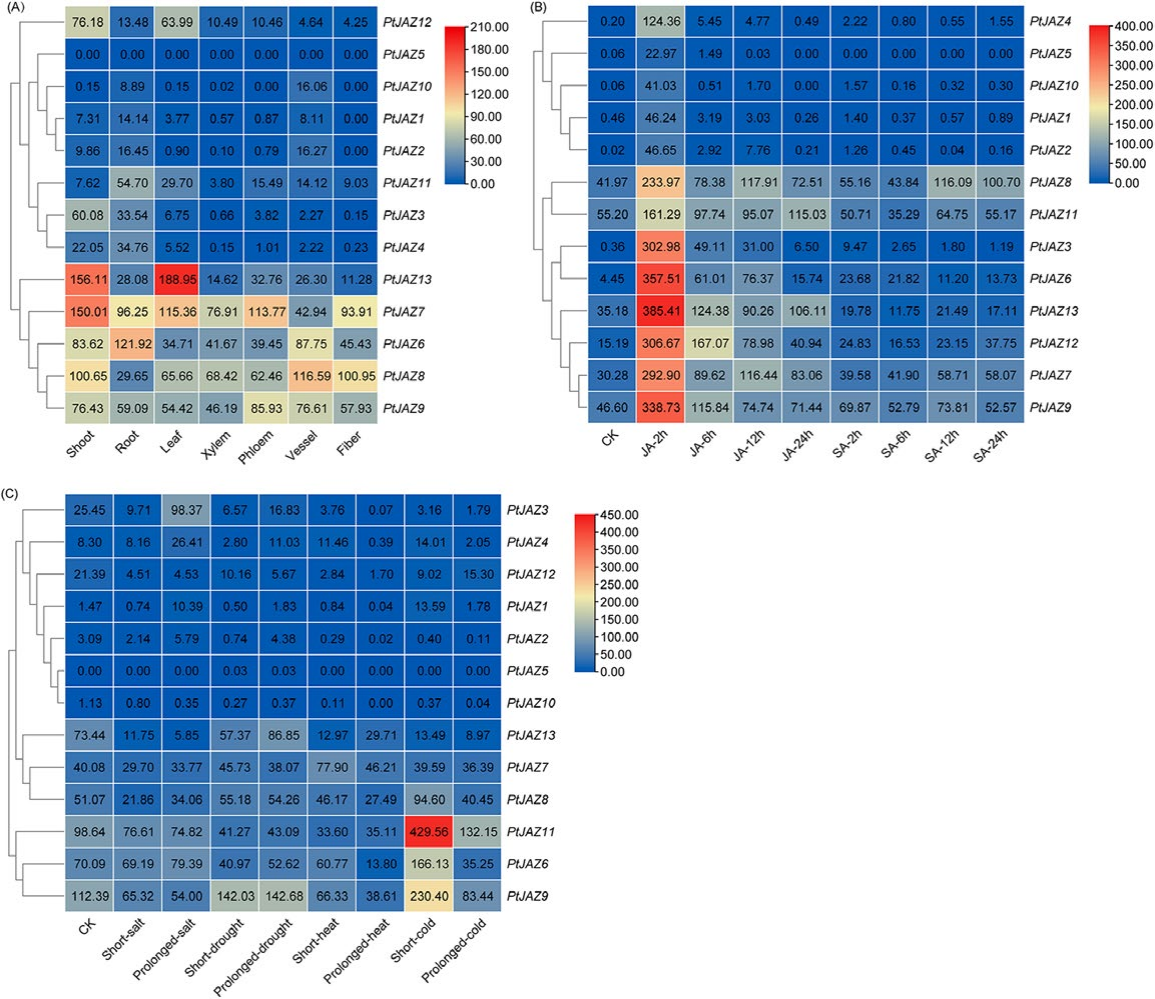
Expression profiles of *PtJAZ* family genes in different tissues and under abiotic stress conditions. (**A**) Expression profiles of *PtJAZ* family genes in different tissues: shoots, roots, leaves, xylem, phloem, vessels and fibers. (**B**) Expression profiles of *PtJAZ* family genes subjected to JA and SA hormone stress treatments. (**C**) Expression profiles of *PtJAZ* family genes under salt, drought, high- and low-temperature stress treatments

Expression pattern analysis can help predict the biological function of genes; therefore, we analyzed *PtJAZ* genes in various tissues (shoot, root, leaf, xylem, phloem, vessel, and fiber) and under abiotic stress (JA, SA, salt, drought, high temperature, and low temperature) through public transcriptome data. As shown in Fig. 7A, the expression patterns of *PtJAZ* genes are different in different tissues. The expression levels of the *PtJAZ1*-*5*, and *PtJAZ10*-*12* genes were relatively lower in all tissues compared to those of the *PtJAZ6*-*9* genes. Among the latter, the expression level of the *PtJAZ6* gene was higher in roots and vessels, and the expression level of the *PtJAZ7* gene was higher in shoots, leaves, and phloem. The expression levels of the *PtJAZ12* and *PtJAZ13* genes were higher in shoots and leaves and lower in other tissues. Figure 7B shows the expression levels under SA and JA hormone stress treatments. Compared with the control, the expression level of the *PtJAZ* gene in poplar under JA stress treatment was higher than that under SA stress treatment. In addition, the expression levels of the *PtJAZ3*, *PtJAZ6*-*9*, and *PtJAZ11*-*13* genes were highest at 2 h after JA treatment. These results indicated that JA treatment induced the expression of these *JAZ* genes. As shown in Fig. 7C, the expression levels of the *PtJAZ1*-*5*, *PtJAZ10*, and *PtJAZ12* genes under salt, drought, high-temperature, and low-temperature stress treatments were low and basically unchanged. The *PtJAZ6*, *PtJAZ9*, and *PtJAZ11* genes were upregulated by short-term cold stress, with the *PtJAZ11* gene showing the most upregulation.

### Expression patterns of *PtJAZ* family genes under biotic stress

**Fig. 8:**
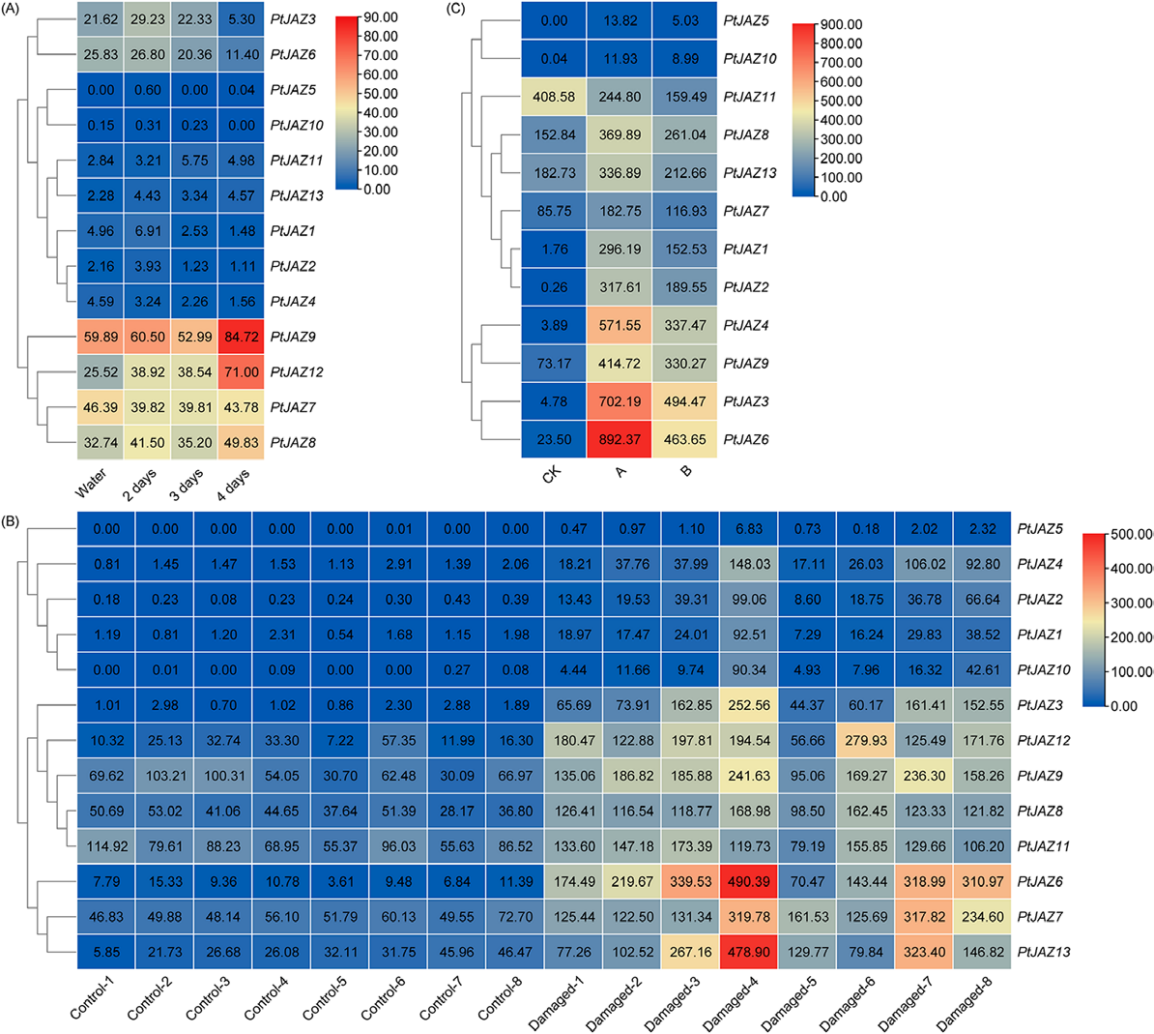
Expression profiles of *PtJAZ* genes under pathogen and insect pest treatments. (**A**) Expression profiles of *PtJAZ* genes under pathogen treatment. (**B**) Expression profiles of *PtJAZ* genes under brassy willow beetle (*Phratora vitellinae*) treatment. (**C**) Expression profiles of *PtJAZ* genes in poplar leaves fed on by *Hyphantria cunea*

We analyzed the expression patterns of *PtJAZ* family genes under biotic stress (pathogen and pest) conditions using public transcriptome data. Figure 8 A shows that the expression levels of *PtJAZ9* and *PtJAZ12* were highly induced after treatment with pathogens for 4 days, indicating that these two genes may be important for regulation of the pathogen response. Figure 8B and C show the transcript abundance of *PtJAZ* family genes when *Phratora vitellinae* and *Hyphantria cunea* feed on poplar leaves, respectively. The two figures show that *PtJAZ3*, *PtJAZ4*, *PtJAZ6*-*9*, and *PtJAZ13* were significantly upregulated after being induced by insects, with the expression of the *PtJAZ6* gene increasing most significantly. Therefore, the *PtJAZ6* gene may play an important role in the pest response.

### Weighted gene coexpression network analysis of *PtJAZ* genes involved in pest stress

**Fig. 9:**
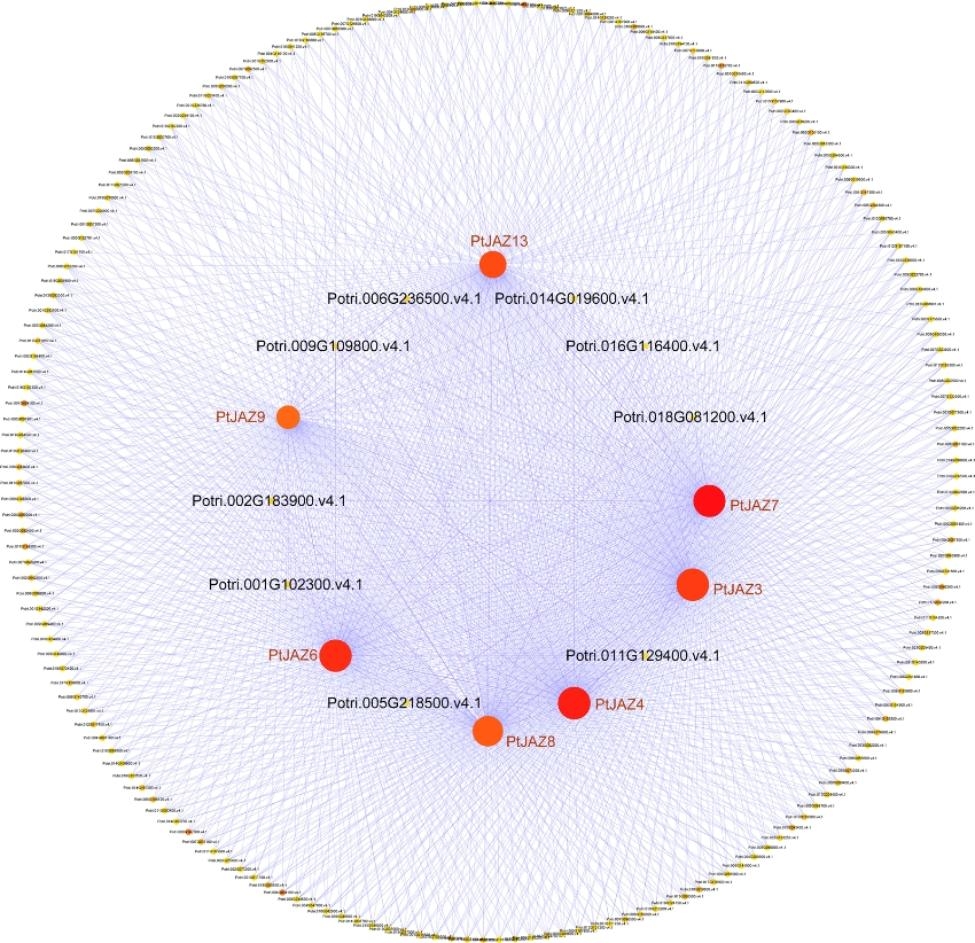
Coexpression network of *PtJAZ* genes in the insect pest feeding response

To explore the potential interactions and functions of coexpressed genes, a coexpression network was constructed based on the transcriptome data of poplar leaves fed on by *Phratora vitellinae*, and Cytoscape software was used for visual analysis. Figure 9 shows that 193 genes were coexpressed with 7 *PtJAZ* genes, including *PtJAZ3*, *PtJAZ4*, *PtJAZ6*, *PtJAZ7*, *PtJAZ8*, *PtJAZ9*, and *PtJAZ13*. These results indicate that the *PtJAZ* family genes may interact with each other and with other genes and participate in the regulatory network regulating pest-induced stress.

### GO and KEGG analysis of genes in the coexpression network of pest stress

**Fig. 10:**
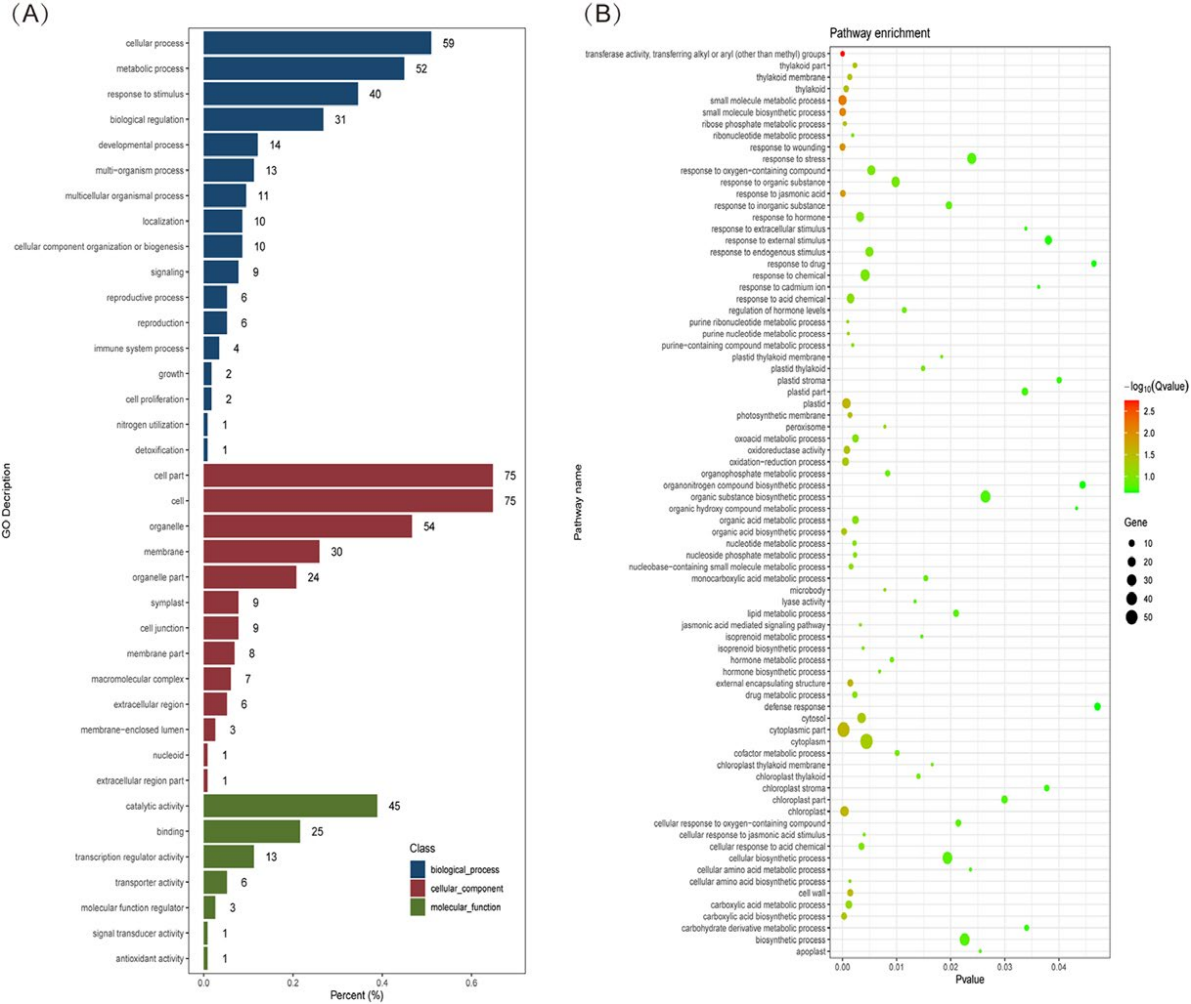
GO and KEGG analysis of genes in the coexpression network. (**A**) GO functional annotation of genes in the coexpression network. (**B**) KEGG pathway enrichment analysis of genes in the coexpression network

The genes in the coexpression network were compared with the Gene Ontology (GO) database, and the results showed that these genes were annotated in 37 GO categories, including 17 for biological processes, 13 for cellular structure, and 7 for molecular functions (Fig. 10A). Among biological processes, the genes were primarily involved in cellular processes, metabolic processes, stimulus responses, and biological regulation. Among the cellular components, the main categories were cell components, cells, organelles, membranes, and membrane components. Among the molecular functions, the main categories were catalytic activity, binding, and transcriptional regulatory activity. These genes were subjected to KEGG pathway analysis through alignment with the KEGG database. The results showed (Fig. 10B) that these genes were significantly enriched in injury response, transferase activity, small molecule metabolic processes, small molecule biosynthesis processes, jasmonate response, cytoplasmic part, carboxylic acid biosynthesis, and a series of related plant insect-resistance relevant pathways.

## Discussion

JA signaling is a key developmental and defense mediator that arose during the transition of plants from aquatic environments to terrestrial environments. JAZ evolved from the *ZIM* gene of the TIFY family through changes in several key amino acids [21]. The JAZ protein was originally isolated and identified from *Arabidopsis thaliana* [22], and it plays a key inhibitory role in the JA signal transduction pathway and has multiple functions.

### Genome-wide identification of the plant *JAZ* gene family

In this study, a total of 13 *PtJAZ* genes were identified. The predicted signal peptides and transmembrane regions were absent; they are nonsecreted proteins rather than membrane proteins. The subcellular localization is predicted to be in the nucleus, which is consistent with the subcellular localization of *NtJAZ1* reported by Panting et al. [23]. Wang et al. [3] studied the poplar *TIFY* gene family in 2017 and identified 24 *TIFY* genes, which they divided into 4 subfamilies, including ZML, JAZ, PPD, and TIFY, of which the JAZ subfamily had 12 members, *PtJAZ1*-*PtJAZ12*. This is consistent with the results identified by Xia et al. [7]. This study identified one more *JAZ* gene, *PtJAZ5* (Potri.006G023301), than that of Wang and Xia et al. [3, 7], probably due to the different methods used to identify gene family members. Wang et al. [3] used *JAZ* from other species to identify homologous genes within the poplar genome. The alignment identified in this study was based on the hidden Markov model of the TIFY and Jaz domains searched by HMMER. Xia et al. [7] used genome annotation version 3.0 of *P. trichocarpa*, and this study used version 4.1 of *P. trichocarpa* genome annotation, which may have contributed to the discrepancy in numbers.

In this study, the *PtJAZ* gene family was divided into four subfamilies, of which the number of subfamilies II was the least and the numbers of subfamilies III and IV were the most. Wang et al. divided the *JAZ* subfamilies in the *TIFY* gene family into six groups (JAZ I-JAZ VI). There are 4 *PtJAZ* genes in JAZ I, the largest group; there is no *PtJAZ* gene in the JAZ V group; and JAZ VI contains only one, *PtJAZ9* [3]. In the classification of family members, the different classifications may be related to the species selected for phylogenetic analysis; for example, the species selected when constructing the phylogenetic tree in this paper are more *Zea mays*, *Juglans regia*, and *Picea sitchensis* than Wang et al., but less *Vitis vinifera* and *Malus pumila Mill* [3]. It may also be related to the choice of software or the subjective choice of classification.

Wang et al. [3] found that most *PtJAZ* genes have 5 to 7 exons, except for *PtJAZ9*, which has only 2 exons. Our study concluded that *PtJAZ* genes have 2 to 7 exons, with *PtJAZ10* in subgroup IV containing 2 exons and all members of subfamily III containing 7 exons, resulting in structural differences, but the differences are not significant, indicating that they have a relatively recent evolutionary relationship. The gene structure and conserved motifs of *BrrJAZ* are similar within subfamilies and all contain conserved TIFY or Jas domains [24]. TIFY and Jas conserved structures are found in the genomes of *Triticum aestivum L.* [25] and *Pinus tabulaeformis* [26]. The C-terminal and N-terminal of the CsJAZ protein contain Jas and TIFY domains, respectively, suggesting that different members of the *CsJAZ* gene family may be involved in different abiotic stresses [27]. In this study, all *PtJAZ* members also contain two typical Jaz and TIFY domains, which are presumed to perform functions similar to those of other plant JAZ proteins.

The *CsJAZ* protein was more similar to the *JAZ* protein in poplar, *Arabidopsis thaliana*, *Vitis vinifera*, and *Gossypium spp*. than to that in *Oryza sativa L.* [9], which is consistent with the fact that the former plants and *Camellia sinensis* are dicotyledons. The collinearity analysis of poplar and other species was not carried out in the two papers by Wang et al. and Xia et al. [3, 7]. In this study, compared with *Arabidopsis thaliana*, *Oryza sativa L.*, and *Eucalyptus*, the number of gene homologous pairs (13) was the highest in *P. trichocarpa* and *Eucalyptus*, suggesting that *P. trichocarpa* and *Eucalyptus* have higher homology and a closer evolutionary relationship. This may be because *P. trichocarpa* and *Eucalyptus* belong to woody plants and are closer relatives than the annual herb *Arabidopsis thaliana*.

### Functional prediction of the *PtJAZ* gene family in poplar

To further understand the function of the *PtJAZ* genes, we analyzed the composition of *cis*-acting elements in *PtJAZ* promoters and the expression of the *PtJAZ* genes in different tissues and under various stress conditions. Previous studies have shown that *cis*-acting elements are important molecular switches in the transcriptional regulation of genes under abiotic or biotic stress [28]. Wang et al. [3] analyzed the *cis*-elements in the promoter region of *PtJAZ* and found that *JAZ12* has up to 12 drought-responsive elements (S000413), *JAZ7* has up to 10 cold-responsive elements (S000407), and *JAZ3* has up to 9 salt response elements (S000453), indicating that the *PtJAZ* gene may be associated with plant stress resistance. In this study, we identified hormone response elements such as ABA, MeJA, GA, IAA, and SA response elements and stress response elements such as low temperature, drought, and resistance response elements in the promoter region of the *PtJAZ* gene. This further revealed that the *PtJAZ* gene may be involved in regulating various stress resistance responses in poplar. The *Pnjaz1*-mediated salt stress tolerance is related to the ABA signal, the MeJA signal, and osmotic pressure [29]. This study found that the *cis*-acting elements of the promoter of the *PtJAZ* gene had the largest number of ABA and MeJA response elements, indicating that the *PtJAZ* gene may be involved in regulating *P. trichocarpa* stress resistance through ABA and MeJA signaling pathways.

Expression profiling analysis in tea plants showed that all 12 *CsJAZ* genes were widely expressed in plant tissues [27]. QRT-PCR (real-time quantitative polymerase chain reaction) analysis showed that seven *CsJAZ* genes were preferentially expressed in roots [8]. The expression trends of *TaJAZ* family member genes in wheat differ in different developmental stages of wheat [25]. Wang et al. [30] conducted expression profiling analysis and showed that *AetJAZ1* and *AetJAZ2* had clear tissue specificity, being specifically expressed only in the panicle, pistil, or stamen. In this study, the *PtJAZ6* gene was highly expressed in roots and vessels, and the *PtJAZ7* gene was highly expressed in shoots, leaves, and phloem. The expression levels of *PtJAZ12* and *PtJAZ13* genes are also high in shoots and leaves, indicating that *PtJAZ* genes are similar to those of other plants and have different expression specificities between different tissues.

In this study, *PtJAZ6*-*9* genes were higher in all tissues; *PtJAZ6*, *PtJAZ9*, and *PtJAZ11* genes were upregulated under low-temperature stress treatment; and the expression levels of *PtJAZ9* and *PtJAZ12* were higher after bacterial stress treatment. Analysis of the transcript abundance of *PtJAZ* family genes in poplar leaves fed on by pests revealed that *PtJAZ6* gene expression was most significantly increased, indicating that the *PtJAZ6* gene plays an important role in insect resistance. Xia et al. [7] showed that the expression of eight genes (*PtJAZ1*, *2*, *4*, *6*, *7*, *9*, *11*, and *12*) was up-regulated after JA treatment for 2 h via qRT-PCR expression profile analysis. The expressions of four genes (*PtJAZ3*, *5*, *9*, and *10*) were up-regulated after SA treatment for 24 h, and the relative expressions of *PtJAZ2*, *3*, *6*, *9*, and *12* in the leaves of “NL895” were the highest after 8 days of inoculation with *M. larici-populina*. The results showed that the relative expression of *PtJAZ9* (corresponding to *PtJAZ7* in this paper) was better [7]. Wang et al. [3] performed qRT-PCR expression profile analysis and showed that *PtJAZ8* (corresponding to *PtJAZ9* in this paper) was highly expressed in all tissues. Five genes (*PtJAZ2*, *3*, *4*, *5*, and *9*) were upregulated after JA treatment. The *PtJAZ3* and *PtJAZ5* genes were upregulated under cold stress, and the *PtJAZ2*, *3*, *4*, and *9* genes were upregulated the most under salt stress, indicating that *PtJAZ3* (corresponding to *PtJAZ3* in this paper) always had the highest expression level, in contrast to this study showing that the *PtJAZ6* gene had the highest level [3]. Wang et al. [3] focused on analyzing the expression patterns of the *PtJAZ* gene under JA, cold stress, and salt stress treatments. Xia et al. [7] focused on analyzing the expression patterns of the *PtJAZ* gene under JA, SA, and pathogen treatment. This study included not only the above four treatments but also drought, high temperatures, and insect pest stress. Considering all of these conditions, *PtJAZ6* may be an important candidate gene for growth and development and the response to stress, but its function needs to be further verified.

Coexpression network analysis is a powerful means of identifying highly correlated gene clusters and predicting gene functions and functional modules [31]. In the coexpression network, we found that there may also be interactions among JAZ proteins. Chini et al. [32, 33] showed that JAZ proteins can form homodimers and heterodimers, so PtJAZ proteins may also be involved in the regulation of insect resistance by forming homodimers or heterodimers. Whether dimers are formed can be verified by subsequent experiments, such as yeast two-hybrid, bimolecular fluorescence complementation, and luciferase complementation imaging. This is the shortcoming of the study by Wang et al. and Xia et al. [3, 7]. This study analyzed the *PtJAZ* co-expression network and concluded that some co-expressed genes were closely related to injury response, stress response, defense response, hormone synthesis, and metabolism, further demonstrating the important role of the *PtJAZ* gene in regulating insect stress response.

## Conclusion

In this study, 13 members of the *JAZ* gene family were identified in poplar using bioinformatics methods and found to be located in the nucleus. The phylogenetic tree divided the 13 *JAZ* genes into 4 subfamilies (subfamilies I-IV), and collinearity analysis suggested that the *JAZ* genes in poplar and *Eucalyptus* have a more recent evolutionary relationship. The analysis of *cis*-regulatory elements lays the foundation for further research on the function of JAZ family genes in response to biotic or abiotic stresses. Transcriptome data analysis showed that different *PtJAZ* genes exhibited different expression patterns. The coexpression network and GO and KEGG analyses indicated that JAZ family genes may participate in the regulatory network of pest-induced stress by interacting with other genes. Therefore, it is speculated that *PtJAZ* may play a key role in plant growth and development and stress response. This study provides a foundation for further research on the function of the poplar *JAZ* genes and may serve as a reference for poplar genetic engineering breeding.

## Materials and methods

### Identification of *JAZ* gene family members in *Populus trichocarpa*

Version 4.1 of *P. trichocarpa* genome data and genome annotation files were downloaded from the Phytozome database (https://phytozome.jgi.doe.gov/). Hidden Markov models of the TIFY (also known as ZIM) (PF06200) and Jaz (also known as CCT_2) (PF09425) domains were constructed using the Pfam database (https://pfam.xfam.org/) [34]. Poplar whole genome protein sequences were searched for potential PtJAZ proteins using HMMER 3.0 software [35]. The domains of the protein sequences were verified using the Pfam tool and CD-search (https://www.ncbi.nlm.nih.gov/Structure/cdd/wrpsb.cgi). In addition, 12 *Arabidopsis thaliana*, 15 *Oryza sativa*, 6 *Zea mays*, 17 *Juglans regia*, and 13 *Picea sitchensis* JAZ protein sequences were obtained from the TAIR database (http://www.arabidopsis.org/), TIGR database (http://rice.plantbiology.msu.edu/), JGI database (https://genome.jgi.doe.gov/), and NCBI database (https://www.ncbi.nlm.nih.gov/).

### Analysis of JAZ gene structure, protein sequence, and promoter *cis*-acting elements in *P. trichocarpa*

The *JAZ* gene structure was analyzed using GSDS 2.0 (Gene Structure Display Server, http://gsds.gao-lab.org/). [36]. The secondary structure of the protein was predicted using the SOPMA online website (https://npsa-prabi.ibcp.fr/cgi-bin/npsa_automat.pl?page=/NPSA/npsa_sopma.html). The subcellular localization of the protein was analyzed using the CELLO v2.5 subcellular localization prediction tool (http://cello.life.nctu.edu.tw/) [37]. Signal peptides were predicted using the SignalP3.0 Server (http://www.cbs.dtu.dk/services/SignalP). Protein transmembrane domains were analyzed using TMHMM (http://www.cbs.dtu.dk/services/TMHMM/). Protein motifs were analyzed using MEME online software (https://meme-suite.org/meme/tools/meme). [38]. The 2000-bp upstream promoter sequences of the *JAZ* genes were extracted from the *P.trichocarpa* genome and submitted to the PlantCARE online database (http://bioinformatics.psb.ugent.be/webtools/plantcare/html/) to analyze promoter *cis*-acting elements [39].

### Multiple sequence alignments and phylogenetic tree construction

Multiple sequence alignments of *P. trichocarpa* JAZ proteins were performed using DNAMAN software. Multiple sequence alignment analysis of JAZ protein sequences of *P. trichocarpa*, *Arabidopsis thaliana*, *Oryza sativa*, *Zea mays*, *Juglans regia*, and *Picea sitchensis* was performed using ClustalW [40]. The phylogenetic tree was constructed using the biological evolution distance method (Neighbor-Joining, NJ) in MEGA 5.0 software [41], and the bootstrap value was 1000.

### Chromosomal location and collinearity analysis

MapChart software [42] was used to draw a draft of the chromosomal locations of the *PtJAZ* family members. The genome sequence files of *P. trichocarpa*, *Arabidopsis*, *Oryza sativa*, and *Eucalyptus robusta* were aligned and analyzed using the One Step MCScanX-Super Fast tool of TBtools software [43]. Based on the comparison results, MCScanX software was used for collinearity mapping; the default parameters were used.

### Expression pattern analysis and weighted gene coexpression network construction

The public RNA-seq data of various *P. trichocarpa* tissues (shoot, root, leaf, xylem, phloem, vessel, and fiber) (accession number: SRX1740285-SRX1740305), different hormone treatments (final concentrations of JA and SA at 0.2 mM and 0.5 mM for samples collected at 2, 6, 12, and 24 h after treatment of fast-growing “NL895” *Populus nigra* × *Populus euramericana*) (accession number: SRX5181815-SRX5181841), abiotic stresses (salt: short-term salt stress of 100 mM NaCl solution for 24 h; long-term salt stress, 100 mM NaCl solution was added to the soil every 2 days, and samples were taken after 7 days. drought: sampling was taken 5 days after the short-term drought cut-off and 12 days after the long-term drought cut-off. high temperature: in a short-term high temperature, the temperature rose to °C 12 h after sampling. Long-term high temperature: the temperature rises to 39 °C 7 days after sampling. low temperature: for short-term low temperature, the temperature is lowered to 12 °C in the light period and 4 °C in the dark period, and the temperature is lowered for 24 h after sampling. Long-term low temperature, lower temperature 7 days after sampling.) (accession number: ERR1864411-ERR1864437), pathogen treatments (*A.alternata* impregnated leaves were treated for 2, 3, and 4 days, accession number: SRR12371687-SRR12371698), and willow beetle treatments (healthy plants from each of the eight asexual poplar lines were selected for willow beetle feeding experiments) (accession number: SRR8424223-SRR8609265) were used for transcript expression analysis. All raw transcriptome sequencing data were downloaded from NCBI (National Center for Biotechnology Information, https://www.ncbi.nlm.nih.gov/). The transcriptome data of the leaves of *P. euramericana* fed on by *Hyphantria cunea* were obtained by our group (*Hyphantria cunea* conducted insect feeding experiments. After feeding insects for 2 h, samples were taken, and leaves with leaf surface loss of about 1/4 were treated as A, and leaves with leaf surface loss of about 1/8 were treated as B (unpublished). TopHat was used to align RNA-seq reads to the reference genome, and the Cufflinks package was used for differentially expressed gene analysis with fragments per kilobase million (FPKM) [44]. The heatmaps of gene expression were visualized using the TBtools program [43]. Using the transcriptome data from the pest treatments noted above, the R/WGCNA package was used to construct a coexpression network [45], and Cytoscape software [46] was used for visual analysis. Gene Ontology (GO) and Kyoto Encyclopedia of Genes and Genomes (KEGG, https://www.kegg.jp/) [47] enrichment analyses were performed on the genes in the coexpression module.

### Electronic supplementary material

Below is the link to the electronic supplementary material.

Supplementary Material 1

## Data Availability

The public RNA-seq data of variou*s Populus trichocarpa* tissues (accession number: SRX1740285-SRX1740305), different hormone treatments (accession number: SRX5181815-SRX5181841), abiotic stresses (accession number: ERR1864411-ERR1864437), pathogen treatments (accession number: SRR12371687-SRR12371698), and willow beetle treatments (accession number: SRR8424223-SRR8609265) The above raw transcriptome sequencing data were downloaded from NCBI (National Center for Biotechnology Information, https://www.ncbi.nlm.nih.gov/). The transcriptome data of *Populus × euramericana* leaves feeding on *Hyphantria cunea* were measured by Dr. Xiaoyue Yu, a senior member of our research group, with whom we agreed to provide the original data. Part of the data has been provided in the supplementary file; the original data is now in the hands of Yu Xiaoyue and Yang Minsheng, the heads of the research team.

## Abbreviations

JAZ: Jasmonate ZIM⁃domain
ABA: Abscisic acid
MeJA: Methyl jasmonate
JA: Jasmonic acid
JA-Ile: Jasmonic acid-amino acid
SA: Salicylic acid
GO: Gene Ontology
KEGG: Kyoto Encyclopedia of Genes and Genomes
GA: Gibberellin
qRT‒PCR: Real-time quantitative polymerase chain reaction

## References

[1] Mao J, Xiong X, Lu Y (2021). Advances in the regulation of plant stress response by jasmonic acid. Chin J Bioprocess Eng.

[2] Huang Z, Wang Z, Li X (2021). Genome-wide identification and expression analysis of *JAZ* gene family in foxtail millet. J Shanxi Agricultural University(Natural Sci Edition).

[3] Wang Y, Pan F, Chen D (2017). Genome-wide identification and analysis of the *Populus trichocarpa TIFY* gene family. Plant Physiol Biochem.

[4] Huang S, Wang H, Fan R (2022). Bioinformatics and expression pattern analysis of the *JAZ* gene family in tomato. Fenzi Zhiwu Yuzhong (Molecular Plant Breeding).

[5] Jia Y. Functional study of Tomato *JAZ10* and *JAZ11* genes in Senescence and Abiotic stresses. Chongqing University; 2020.

[6] Xia J, Jia D, Zeng J, et al. Cloning and functional analysis of *JAZ* genes in Artemisia annua Acta Pharmaceutica Sinica. 2018; 53(05):812–8.

[7] Xia W, Yu H, Cao P, Luo J, Wang N (2017). Identification of TIFY family genes and analysis of their expression profiles in response to phytohormone treatments and melampsora larici-populina infection in poplar. Front Plant Sci.

[8] Zhai Z. Transcriptome profiling of Aegilops tauschii Leaves by Jasmonate treatments and characterization of JAZ Family genes. Chinese Academy of Agricultural Sciences; 2018.

[9] Shen J, Zou Z, Xing X (2020). Genome-wide analysis reveals stress and hormone responsive patterns of JAZ Family genes in *Camellia Sinensis*. Int J Mol Sci.

[10] Huang Z, Wang Z, Li X (2021). Genome-wide identification and expression analysis of JAZ family involved in hormone and abiotic stress in sweet potato and its two diploid relatives. Int J Mol Sci.

[11] Sun Y, Liu C, Liu Z (2021). Genome-wide identification, characterization and expression analysis of the *JAZ* gene family in resistance to gray leaf spots in tomato. Int J Mol Sci.

[12] Han Y, Luthe D (2021). Identification and evolution analysis of the *JAZ* gene family in maize. BMC Genomics.

[13] Liu K, Li J, Wang H (2021). Identification and transcriptional expression analysis of walnut *JrJAZ* gene family. J HEBEI AGRICULTURAL Univ.

[14] Li W, Song J, Zhu S, et al. Analysis of the induced response and interaction among tomato JAZ family members to pathogens. Fenzi Zhiwu Yuzhong(Molecular Plant Breeding; 2022.

[15] Zhang Y, GENOME-WIDE IDENTIFICATION AND ANALYSIS OF GRAPE ALDEHYDE. DEHYDROGENASE (ALDH) AND JASMONATE ZIM-DOMAIN (JAZ) GENE FAMILIES. Northwest A&F University; 2012. 10.1371/journal.pone.0032153 PMC3280228 22355416

[16] Li W, Xia X, Han L (2017). Genome-wide identification and characterization of *JAZ* gene family in upland cotton (*Gossypium hirsutum*). Sci Rep.

[17] Wu H. Characterization and function analysis of Rice stress-related *JAZ* genes. Huazhong Agricultural University; 2015.

[18] Sheng Y, Yu H, Pan H (2022). Genome-wide analysis of the Gene structure, expression and protein interactions of the Peach (*Prunus persica*) *TIFY* Gene Family. Front Plant Sci.

[19] Song H, Duan Z, Wang Z (2022). Genome-wide identification, expression pattern and subcellular localization analysis of the *JAZ* gene family in *Toona ciliata*. Ind Crops Prod.

[20] Tuskan G, di Fazio S, Jansson S (2006). Supporting online materialfor the genome of black cottonwood. Populus trichocarpa(Torr &Gray) Science.

[21] Schluttenhofer C (2020). Origin and evolution of jasmonate signaling. Plant Sci.

[22] Wei X, Liu Y, Liu YY (2021). Advances of JAZ family in plants. Plant Physiol J.

[23] Pan T, Hu L, Wang Z (2018). Cloning and function analysis of *JAZ1* gene from *Nicotiana tabacum*. Tob Sci Technol.

[24] Jia K, Yan C, Zhang J (2021). Genome-wide identification and expression analysis of the *JAZ* gene family in turnip. Sci Rep.

[25] Jv L. Molecular mechanism of JAZ Proteins modulating seed germination in Bread Wheat and Arabidopsis. Northwest A&F University; 2020.

[26] Zhang J, Ma Y, Wang H (2022). Characteristic of *JAZ* gene family of Pinus tabuliformis and identification of functional domain of its interaction with DELLA protein. J Beijing Forestry Univ.

[27] Zheng Y, Chen X, Wang P (2020). Genome-wide and expression pattern analysis of JAZ family involved in stress responses and postharvest processing treatments in *Camellia sinensis*. Sci Rep.

[28] Nakashima K, Ito Y, Yamaguchi-Shinozaki K (2009). Transcriptional regulatory networks in response to abiotic stresses in Arabidopsis and grasses. Plant Physiol.

[29] Liu S, Zhang P, Li C, et al. The moss jasmonate ZIM-domain protein *PnJAZ1* confers salinity tolerance via crosstalk with the abscisic acid signalling pathway. Plant Sci. 2019;280:1–11. 10.1016/j.plantsci.2018.11.004 30823987

[30] Wang Y, Yuan S, Yuan G, et al. Genome-wide identification and analysis of *JAZ* Gene Family in Aegilopstauschii. J Triticeae Crops. 2016;36(01):9–17.

[31] Sun H, Chen L, Li J (2017). The JASMONATE ZIM-domain gene family mediates JA signaling and stress response in cotton. Plant Cell Physiol.

[32] Chini A, Fonseca S, Chico J, Fernández-Calvo P, Solano R (2009). The ZIM domain mediates homo‐and heteromeric interactions between Arabidopsis JAZ proteins. Plant J.

[33] Yan S, Sun C, Zhou X (2015). Cloning and characterization analysis of *ZmJAZ4*, a JAZ Family Gene in Maize(*Zea mays L*). Biotechnol Bull.

[34] Sara G, Jaina M, Alex B, Eddy R, Aurélien L, Potter C, Matloob Q, Richardson J, Salazar A, Alfredo S (2019). The pfam protein families database in 2019. Nuclc Acids Research.

[35] Prakash A, Jeffryes M, Bateman A, Finn D (2017). The HMMER web server for protein sequence similarity search. Curr Protocols Bioinf.

[36] Bo H, Pu J, Yuan A, et al. GSDS 2.0: an upgraded gene feature visualization server. Bioinformatics. 2015. 10.1093/bioinformatics/btu817 PMC4393523 25504850

[37] Yu S, Chen C, Lu H, et al. Prediction of protein subcellular localization. Proteins. 2006(3): 643–51. 10.1002/prot.21018 16752418

[38] Bailey L, Boden M, Buske A, Frith M, Grant E, Clementi L, Ren J, Li W, Noble S (2009). MEME SUITE: tools for motif discovery and searching. Nucleic Acids Res.

[39] Magali L, Patrice D, Gert T, Kathleen M, Yves M, Yves P, Pierre R. Stephane R. PlantCARE, a database of plant cis-acting regulatory elements and a portal to tools for in silico analysis of promoter sequences. Nucleic Acids Res. 2002(1): 325–7. 10.1093/nar/30.1.325 PMC99092 11752327

[40] Katoh K, Standley M. MAFFT: iterative refinement and additional methods. Multiple sequence alignment methods. Springer. 2014;131–46. 10.1007/978-1-62703-646-7_8 24170399

[41] Guindon S, Lethiec F, Duroux P, Gascuel O (2005). PHYML Online—a web server for fast maximum likelihood-based phylogenetic inference. Nucleic Acids Res.

[42] MapChart, VOORRIPS R (2002). Software for the graphical presentation of linkage maps and QTLs. J Hered.

[43] Chen C, Chen H, Zhang Y, Thomas R, Xia R (2020). TBtools: an integrative Toolkit developed for interactive analyses of big Biological Data. Mol Plant.

[44] Trapnell C, Goff L, Pertea G, Kim D, Kelley D (2012). Differential gene and transcript expression analysis of RNA-seq experiments with TopHat and Cufflinks. Nat Protoc.

[45] Langfelder P, Horvath S, Langfelder P, Horvath S. WGCNA: an R package for weighted correlation network analysis. BMC Bioinformatics 2008; 9(559). 10.1186/1471-2105-9-559 PMC2631488 19114008

[46] Shannon P, Markiel A, Ozier O, Baliga S, Wang T, Ramage D, Amin N, Schwikowski B, Ideker T (2003). Cytoscape: a software environment for integrated models of biomolecular interaction networks. Genome Res.

[47] Kanehisa M, Goto S (2000). KEGG: Kyoto Encyclopedia of genes and genomes. Nucleic Acids Res.

